# Draft Genome Sequence of Streptomyces albus Strain NBRC 13014^T^, the Type Species of the Genus Streptomyces

**DOI:** 10.1128/genomeA.01527-14

**Published:** 2015-02-05

**Authors:** Hisayuki Komaki, Natsuko Ichikawa, Akio Oguchi, Moriyuki Hamada, Tomohiko Tamura, Nobuyuki Fujita

**Affiliations:** a Biological Resource Center, National Institute of Technology and Evaluation (NBRC), Chiba, Japan; b NBRC, Tokyo, Japan

## Abstract

Streptomyces albus is the type species of the genus Streptomyces. Here, we report the draft genome sequence of S. albus strain NBRC 13014^T^. The genome contains at least seven orphan polyketide synthase and nonribosomal peptide synthetase gene clusters. The genome sequence will also serve as a valuable reference for Streptomyces taxonomy.

## GENOME ANNOUNCEMENT

Members of the genus Streptomyces are promising sources for secondary metabolites (1). Streptomyces albus, previously identified as S. albus subsp. albus, is the type species of the genus Streptomyces (2). Some members of S. albus are reported to be industrially useful strains; S. albus ATCC 21838 (DSM 41398) is known as a salinomycin producer (3, 4), and S. albus J1074 is used for a host for heterogeneous gene expression. A S. albus J1074 genome sequence was recently published (5), but it was reported that this strain was not a member of S. albus (2). Despite the increasing number of recent Streptomyces genome-sequencing projects (6, 7), the genome sequence of the type strain of S. albus had not been available when we started this study. Hence, we performed whole-genome shotgun sequencing of S. albus NBRC 13014^T^.

The whole genome of S. albus NBRC 13014^T^ was read by using a combined strategy of shotgun sequencing with GS FLX+ (Roche; 75.7-Mb sequences, 9.7-fold coverage) and paired-end sequencing with MiSeq (Illumina; 513.8-Mb sequences, 66.1-fold coverage). The reads were assembled using the Newbler version 2.6 software and subsequently finished using a GenoFinisher software (8), which led to a final assembly of 95 scaffold sequences of >500 bp each. The total size of the assembly was 7,594,701 bp, with a G+C content of 72.7%. Coding sequences were predicted by Prodigal version 2.6 (9). Polyketide synthase (PKS) and nonribosomal peptide synthetase (NRPS) gene clusters were determined as previously reported (10). The genome contained at least four type-I PKS, one type-II PKS, two NRPS, and two hybrid PKS/NRPS gene clusters. The type-II PKS gene cluster will synthesize xantholipin-like compounds because its KSα and CLF showed 89% and 78% amino acid sequence identities to XanF and XanE, respectively (11). This strain did not possess a salinomycin-biosynthetic gene cluster (3, 4).

More recently, the draft genome sequence of S. albus subsp. albus NRRL B-1811^T^ (GenBank accession no. JODR00000000.1) was released to the public. In the draft genome sequence, two small type-I PKS gene clusters were completely sequenced, but large type-I PKS gene clusters were divided into 12 short contigs. In contrast, we revealed that S. albus NBRC 13014^T^ harbors at least two large type-I PKS gene clusters, one of which has more than 17 modules, and another of which has over six modules. Therefore, the NBRC 13014^T^ draft genome sequence can provide better information to analyze the type-I PKS gene clusters.

The genome sequence reported here will facilitate studies into the elucidation of the potential of this species as sources of bioactive secondary metabolites and serve as a valuable reference for taxonomic studies of the genus Streptomyces.

### Nucleotide sequence accession numbers.

This draft genome sequence of S. albus NBRC 13014^T^ has been deposited in DDBJ/ENA/GenBank under the accession number BBQG00000000. The version described in this paper is the first version, BBQG01000000.
